# Systematic Identification and Analysis of Circular RNAs of Japanese Flounder (*Paralichthys olivaceus*) in Response to *Vibrio anguillarum* Infection

**DOI:** 10.3390/genes12010100

**Published:** 2021-01-15

**Authors:** Xianhui Ning, Li Sun

**Affiliations:** 1 CAS Key Laboratory of Experimental Marine Biology, Institute of Oceanology, Center for Ocean Mega-Science, Chinese Academy of Sciences, Qingdao 266071, China; xhningouc@163.com; 2 Laboratory for Marine Biology and Biotechnology, Qingdao National Laboratory for Marine Science and Technology, Qingdao 266237, China

**Keywords:** CircRNA, *Paralichthys olivaceus*, *Vibrio anguillarum*, immune-related networks

## Abstract

Circular RNA (circRNA) is a new class of non-coding RNA that is structured into a closed loop without polyadenylation. Recent studies showed that circRNAs are involved in the host immune response to pathogen infection. Japanese flounder (*Paralichthys olivaceus*), an important economical marine fish cultured in north Asia, is affected by *Vibrio anguillarum*, a pathogenic bacterium that can infect a large number of fish. In this study, we systematically explored the circRNAs in the spleen of *V. anguillarum*-infected flounder at different infection time points. A total of 6581 circRNAs were identified, 148 of which showed differential expression patterns after *V. anguillarum* infection and were named DEcirs. Most of the DEcirs were strongly time-specific. The parental genes of the DEcirs were identified and functionally classified into diverse pathways, including immune-related pathways. Among the immune-related DEcirs, seven were predicted to sponge 18 targeted miRNAs that were differentially expressed during *V. anguillarum* infection (named DETmiRs). Further analysis showed that the DEcirs and their corresponding DETmiRs intertwined into complicated immune related networks. These results indicate that in flounder, circRNAs are regulated by *V. anguillarum* and form interactive networks with mRNAs and miRNAs that likely play important roles in the immune defense against pathogen infection.

## 1. Introduction

Circular RNA (circRNA) is a type of non-coding RNA discovered recently. Unlike other RNA molecules with linear structures, circRNA features as a closed loop, formed by linking the 3′ and 5′ ends through covalent bonds [1]. CircRNAs are generated by head-to-tail splicing at the splice sites, where the splicing acceptor site at upstream is covalently bonded to the downstream splicing donor site [2]. The typical regions in the genome that produce circRNAs are protein coding gene sites, including exon and intron sites [3]. Recently, circRNAs produced from intergenic and antisense regions have also been discovered [4]. CircRNAs are widespread in spatial- and temporal-specific patterns [5,6].

The general functions of circRNAs remain largely unknown. Recently, several lines of evidence have indicated that circRNAs play a role in pathogenesis and disease progression by acting as post-transcriptional regulators [7,8,9,10]. CircRNAs can affect parental gene expression directly via specific RNA–RNA interactions [11] and can also serve as microRNA (miRNA) sponges [12]. In fish, several studies on circRNA have been reported. In grass carp, spleen and kidney circRNAs were shown to be associated with grass carp reovirus (GCRV) infection [13,14]. In tilapia, circRNAs modulated host response to *Streptococcus agalactiae* infection [15]. In Japanese flounder, intestinal circRNAs participated in anti-bacteria (*Edwardsiella tarda*) infection [16]. These observations indicate that circRNAs are involved in fish immunity against pathogen infection.

Japanese flounder (*P. olivaceus*) is one of the most important aquaculture species widely cultured in north Asia [17]. The flounder farming industry has been affected by the outbreaks of bacterial diseases, including vibriosis. *V. anguillarum* is a Gram-negative bacterium and an etiological agent of vibriosis [18]. Great efforts have been made to investigate protein-coding genes of flounder against *V. anguillarum* [19,20,21,22]. Recently, immune-related miRNAs of flounder have been studied in association with *V. anguillarum* infection [23]. However, no study on the involvement of circRNAs in flounder immune defense against *V. anguillarum* has been documented.

In this study, we systematically analyzed the spleen circRNAs of flounder in response to *V. anguillarum* infection at three different time points (6 h, 12 h, and 24 h post-infection). We detected the circRNAs and the corresponding parental genes. CircRNAs exhibited differential expressions (DEcirs) after *V. anguillarum* infection were characterized. The parental genes of DEcirs were identified and enriched functionally. Finally, immune related DEcir-miRNA networks were constructed. Our study provides new insights into the mechanism of antibacterial immunity in flounder.

## 2. Materials and Methods

### 2.1. CircRNA Identification

The previously reported RNA-seq data of flounder infected with *V. anguillarum* at 6, 12, and 24 hpi [22] were retrieved. The experimental fish and the procedures of bacterial infection were described in the previous report [22]. Briefly, clinically healthy Japanese flounder with body weight of 214.7 ± 15.2 g were used in the experiment. The fish were maintained at 20 ± 1 °C in aquariums for one week before the experiment. Fish in group V (*V. anguillarum*-infected group) were injected intramuscularly with 200 μL *V. anguillarum* suspension at a concentration of 5 × 10^8^ colony forming units (CFU) mL^−1^, while group C (control group) were injected with the same volume of PBS [22]. This dataset includes 18 libraries consisting of RNAs from fish in control group (named group C) and *V. anguillarum*-infected group (named group V) at 6, 12, and 24 hpi, with three biological repeats at each time point [22]. The high-quality trimmed (HQT) reads were obtained using the programs fastp (v0.12.4) and Bowtie 2 (v2.2.8) as described previously [22]. The HQT reads were then mapped to the reference genome of flounder using tophat 2 (v2.0.3.12) [24]. The reads that aligned contiguously to the genome were removed. The unmapped reads with splice sites were retained and subjected to program find_circ [1] to identify circRNAs. First, the 20 monomers from both ends of the retained reads were extracted and mapped again to the genome to screen the unique anchor positions within the spliced exons. Second, the anchor reads aligned to the genome in the head-to-tail direction were extracted. Third, anchor reads with ambiguous breakpoints or lengths more than 100 kb were discarded. Finally, the anchor reads that aligned in the head-to-tail direction and with unique breakpoints were identified as circRNAs of Japanese flounder.

### 2.2. CircRNA Characterization

The program find_circ (//github.com/marvin-jens/find_circ/archive/v1.2.tar.gz) [1] was used to classify the types (exons, one exon, exon–intron, intronic, antisense, and intergenic) of the identified circRNAs according to the genome regions where the circRNAs generated, and the transcriptome annotation of the reference genome of flounder. Statistical analysis of length distribution was conducted using R (v3.5.2). The abundance of circRNA was calculated and normalized using Reads Per Million mapped reads (RPM) to eliminate the influence on circRNA expression calculation induced by different sequencing libraries.

### 2.3. Functional Enrichment Analysis

GO functional enrichment and KEGG pathway enrichment were performed using the GO database (http://geneontology.org) and KEGG database (http://www.genome.jp/kegg/), respectively. The statistical significance was examined using the hypergeometric test, and *p* < 0.05 was considered the threshold to identify significantly enriched GO terms and KEGG pathways as previously reported [22].

### 2.4. Differential Expression Analysis

Differential expression analysis was performed by pairwise comparison of group C and group V at each time point using the edgeR package (http://www.rproject.org/). The circRNA with a fold change ≥ 2 or ≤ −2 and a *p* value < 0.05 was identified as significantly and differentially expressed circRNA (named DEcir). qRT-PCR was carried out to validate the expression patterns of 6 parental genes of immune-related DEcirs. qRT-PCR was performed with ChamQ SYBR qPCR Master Mix (Vazyme, Nanjing, China) using QuantStudio 3 Real-Time PCR Systems (Thermo Fisher Scientific, San José, CA, USA) according to the manufacturer’s protocol. The expression of each gene was normalized to that of TUBA with 2^−ΔΔCt^ comparative Ct method as reported previously [22]. The primers used for qRT-PCR analysis were listed in Table S1.

### 2.5. Identification of the Target miRNAs of DEcirs

Three programs—Miranda (v3.3a), TargetScan (V7.0), and RNAhybrid (v2.1.2) + svm_light (v6.01)—were used to predict the candidate target miRNAs of DEcirs based on the previous micro-transcriptome dataset of *V. anguillarum*-infected flounder [23]. The miRNAs identified with all three algorithms were considered the candidate target miRNAs, which were then subjected to R (v3.5.2) analysis to detect differentially expressed miRNAs with log_2_|FC| > 1 at all three time points. These differentially expressed miRNAs were considered the target miRNAs of DEcirs and named DETmiRs.

### 2.6. Immune-Related DEcir-miRNA Network Construction

The immune-related DEcirs were obtained base on the KEGG enrichment of their parental genes. The interacting networks of the immune-related DEcirs and the corresponding DETmiRs were constructed using Cytoscape (v3.7.1) [25].

## 3. Results

### 3.1. Characterization of Flounder circRNAs Induced by V. anguillarum

**Figure 1:**
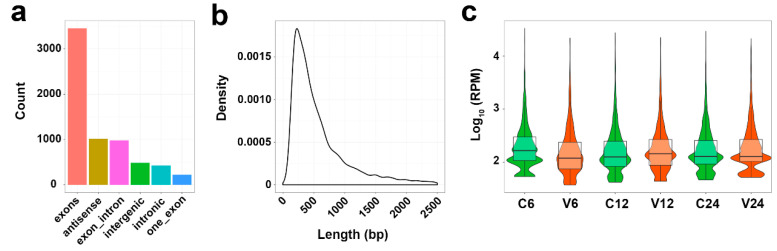
The features of circRNAs in Japanese flounder induced by *V. anguillarum*. (**a**) Classification of circRNAs. (**b**) The length distribution of circRNAs. bp, base pair. (**c**) The expression profiles of circRNAs in different groups at different time points. RPM, reads per million mapped reads. For convenience, “C6”, “C12”, and “C24” indicate the control groups at 6, 12, and 24 h post-infection (hpi), respectively; “V6”, “V12”, and “V24” indicate the *V. anguillarum*-infected groups at 6, 12, and 24 hpi, respectively.

To identify circRNAs in the spleen of flounder induced during *V. anguillarum* infection, we screened the RNA-seq data of *V. anguillarum*-infected flounder reported in a previous study [22]. A total of 6581 circRNAs were identified, which all have anchor reads that aligned in the head-to-tail direction and with unique breakpoints. Based on the generation regions of the circRNAs in the genome, these circRNAs were classified into six types: exons, one exon, exon–intron, intronic, antisense, and intergenic circRNAs (Figure 1a). The lengths of most identified circRNAs were in the range of 201 to 300 nt (Figure 1b). The expression profiles of the circRNAs in the control groups and *V. anguillarum*-infected groups are shown in Figure 1c.

### 3.2. Identification and Functional Enrichment of the Parental Genes of circRNAs

**Figure 2:**
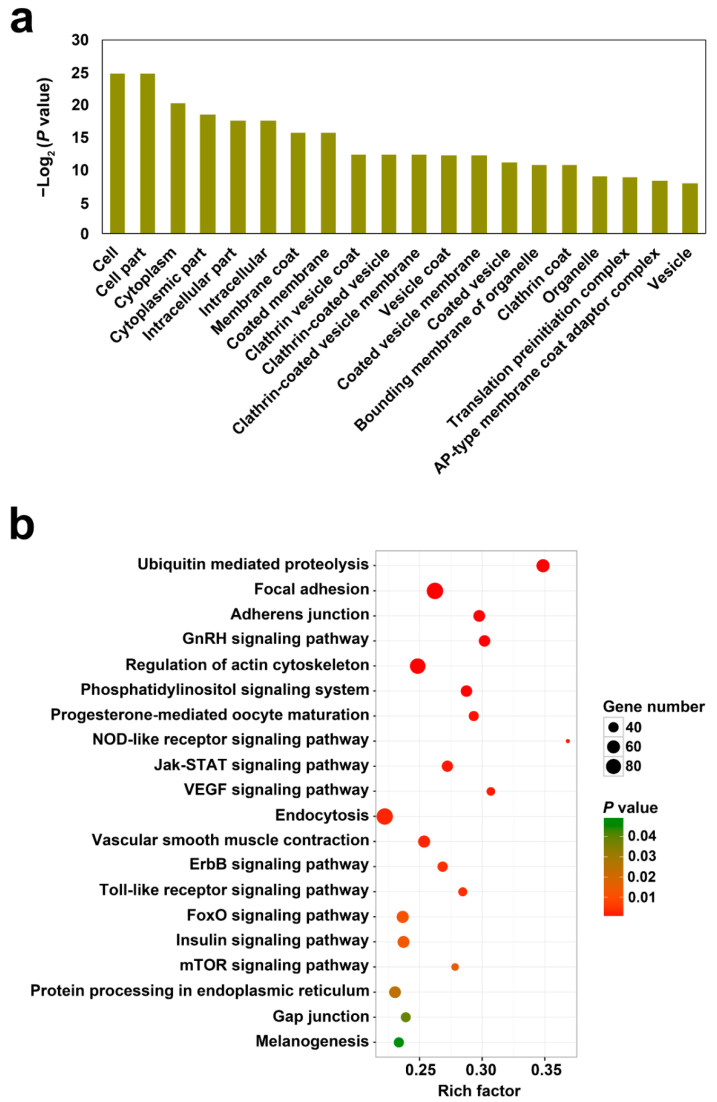
GO (**a**) and KEGG (**b**) functional enrichment of the parental genes of the identified circRNAs in Japanese flounder.

For the 6581 circRNAs, 3396 parental genes were identified in Japanese flounder. GO and KEGG enrichment analyses were applied to analyze the functions of the parental genes. As shown in Figure 2a, the parental genes were classified into various GO functional terms, including cell part, membrane, and vesicles, in particular clathrin associated vesicles. KEGG analysis enriched the parental genes into diverse pathways including those involved in immunity, such as the signaling pathways of NOD-like receptor, Jak-STAT, Toll-like receptor, phosphatidylinositol, ErbB, mTOR, and ubiquitin-mediated proteolysis and endocytosis (Figure 2b).

### 3.3. Identification of Differentially Expressed circRNAs (DEcir) Induced by V. anguillarum

**Figure 3:**
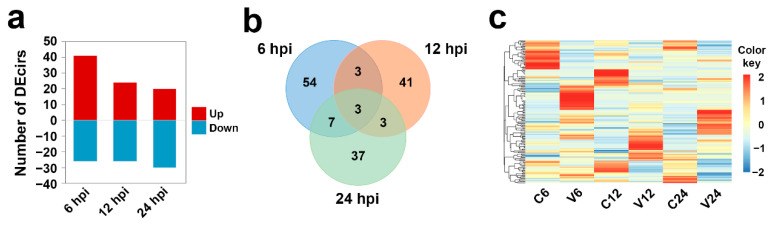
Differentially expressed circRNAs (DEcirs) induced by *V. anguillarum* infection at different time points. (**a**) Number of DEcirs at 6, 12, and 24 h post-infection (hpi). “Up” and “Down” indicate up- and downregulated expression, respectively. (**b**) Venn diagram showing overlapping DEcirs at different time points. (**c**) Heat-map showing the expression patterns of DEcirs in different groups at different time points. For convenience, “C6”, “C12”, and “C24” indicate the control groups at 6, 12, and 24 h post-infection (hpi), respectively; “V6”, “V12”, and “V24” indicate the *V. anguillarum*-infected groups at 6, 12, and 24 hpi, respectively.

After *V. anguillarum* infection, 67 circRNAs showed differential expression patterns at 6 hpi, 41 and 26 of which were up- and downregulated, respectively. Fifty circRNAs showed differential expression at 12 hpi, 24 and 26 of which were up- and downregulated, respectively. Fifty circRNAs showed differential expression patterns at 24 hpi, 20 and 30 of which were up- and downregulated, respectively (Figure 3a). Of the 148 DEcirs identified, only three exhibited differential expression at all the three time points (Figure 3b). As shown in Figure 3c, the expression profiles of all DEcirs were time-dependent.

### 3.4. Identification and Functional Enrichment of the Parental Genes of DEcirs

**Figure 4:**
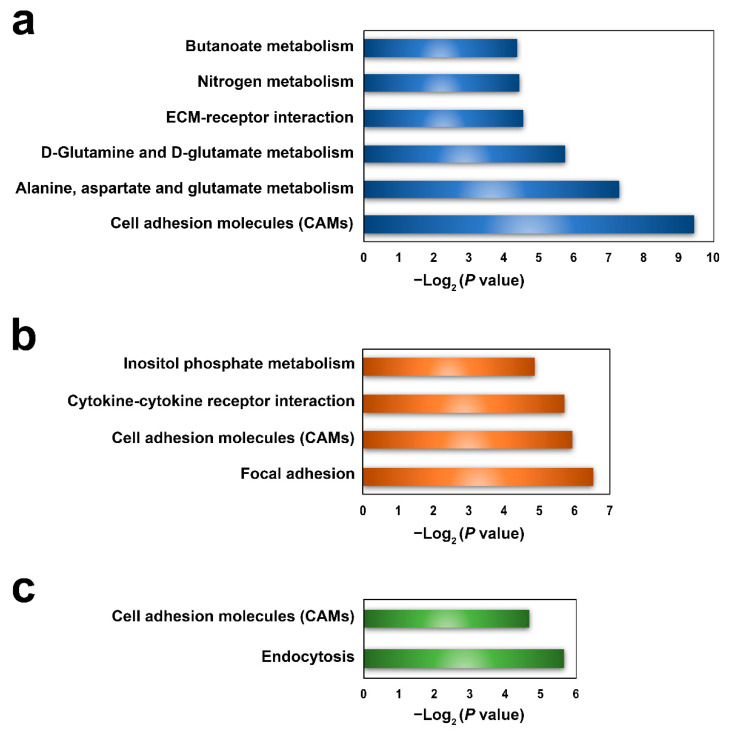
KEGG functional enrichment of parental genes of DEcirs at 6 hpi (**a**), 12 hpi (**b**), and 24 hpi (**c**).

**Figure 5:**
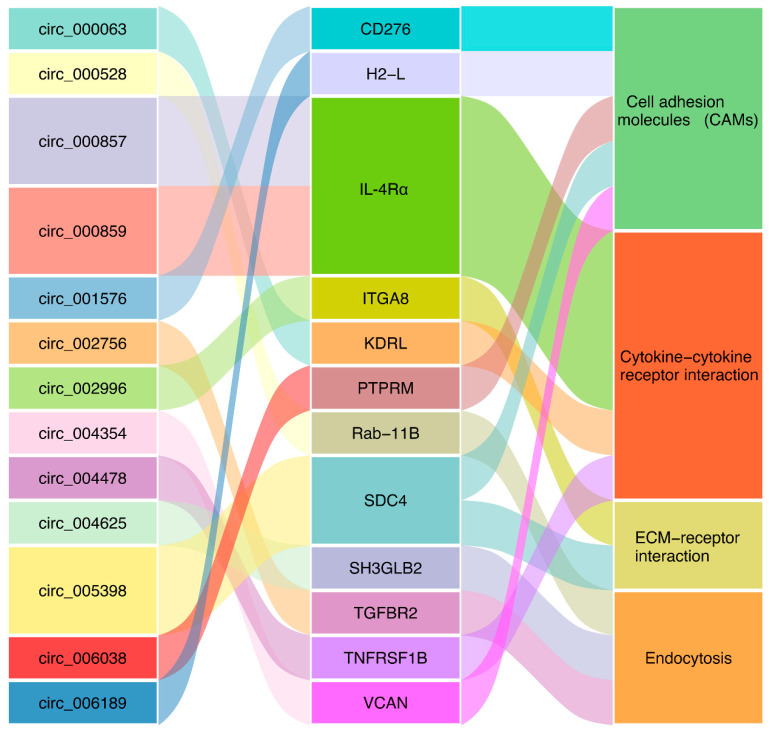
DEcirs and the corresponding parental genes associated with four immune-related pathways. *CD276*, CD276 antigen; *H2-L*, H-2 class I histocompatibility antigen, α chain-like; *IL-4Rα*, interleukin-4 receptor subunit α; *ITGA8*, integrin α 8; *KDRL*, vascular endothelial growth factor receptor kdr-like; *PTPRM*, receptor-type tyrosine-protein phosphatase mu-like; *Rab-11B*, ras-related protein Rab-11B; *SDC4*, syndecan-4-like; *SH3GLB2*, SH3 domain containing GRB2 like, endophilin B2; *TGFBR2*, TGF-β receptor type 2; *TNFRSF1B*, tumor necrosis factor receptor superfamily member 1B; *VCAN*, versican core protein.

A total of 59, 55, and 46 parental genes were identified for the DEcirs at 6 hpi, 12 hpi, and 24 hpi, respectively. KEGG enrichment analysis showed that 6, 4, and 2 pathways associated mainly with metabolism and molecular interactions were significantly enriched at 6 hpi, 12 hpi, and 24 hpi, respectively (Figure 4). Cell adhesion molecules (CAMs), which are involved in immunity, were the common biological process regulated by DEcirs at all three time points (Figure 4). The immune-related pathways of extracellular matrix (ECM)–receptor interaction, cytokine–cytokine receptor interaction, and endocytosis were enriched at 6 hpi, 12 hpi, and 24 hpi, respectively. Based on the enriched immune pathways, 13 immune-related DEcirs and 12 corresponding parental genes were obtained, including the interacting DEcir-parental gene pairs of circ_005398-SDC4 (syndecan-4-like), circ_000857-IL-4Rα (interleukin-4 receptor subunit α), circ_000859-IL-4Rα, and circ_004625-SH3GLB2 (SH3 domain containing GRB2 like, endophilin B2) (Figure 5). The expression patterns of four parental genes of the DEcirs, i.e., IL-4Rα, CD276, H2-L (H-2 class I histocompatibility antigen, α chain-like), and TGFBR2 (TGF-β receptor type 2), at different time points were validated by qRT-PCR. The results showed that the expression patterns of these genes determined by qRT-PCR were similar to that determined by RNA-seq (Figure S1). The expression patterns of two other parental genes, i.e., SDC4 and ITGA8 (integrin α 8), involved in the ECM-receptor interaction pathway were also validated by qRT-PCR (Figure S2).

### 3.5. Immune-Related Networks of DEcirs and Differentially Expressed Target miRNAs of DEcirs (DETmiRs)

**Figure 6:**
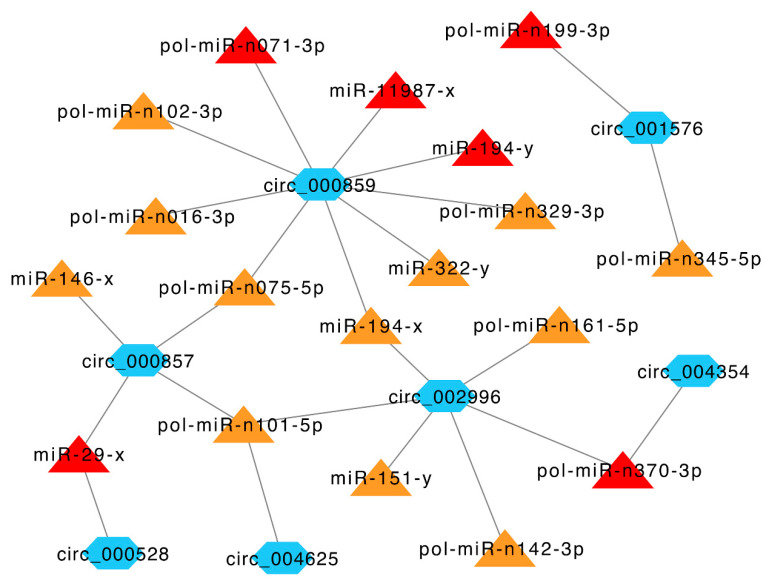
Immune-related networks formed by interactive DEcirs and DETmiRs. The blue hexagon nodes indicate DEcirs. The triangle nodes indicate DETmiRs, of which the key miRNAs induced by *V. anguillarum* identified in a previous study [23] are in red, and the miRNAs identified in this study are in orange. The interactive relationships between DEcirs and DETmiRs are indicated by connecting lines.

Given the broad impact of circRNAs on miRNA activity, we explored the target miRNAs of immune-related DEcirs. Eighteen DETmiRs were identified for seven immune-related DEcirs. Notably, six of the 18 DETmiRs, i.e., pol-miR-n199-3p, pol-miR-n071-3p, pol-miR-n370-3p, miR-11987-x, miR-194-y, and miR-29-x, were key miRNAs identified in a previous miRNA transcriptome analysis of *V. anguillarum*-infected Japanese flounder [23]. To gain insight into the relationships among the DEcirs and DETmiRs, immune-related networks were constructed, which revealed complicated interactions among the DEcirs and DETmiRs (Figure 6).

## 4. Discussion

In this study, we systematically identified the circRNAs in flounder spleen, one of the major immune organs of teleost fish [26], in response to *V. anguillarum* infection at three different time points. Most identified circRNAs in this study were generated from the protein-coding gene regions, which is in line with the observation in grass carp [14]. Moreover, we found that some of the parental genes are involved in various immune-related processes, indicating that circRNAs are regulators of flounder immunity.

Of the 148 DEcirs identified at the three time points of *V. anguillarum* infection, only three circRNAs showed differential expression at all the time points. Moreover, the DEcirs at different time points were engaged in different immune pathways through their parental genes. These results are in accord with the reports that circRNAs exhibit time-specific expression patterns [27,28]. In our study, the ECM–receptor interaction was enriched in the parental genes of DEcirs at 6 hpi. As ECMs have a profound impact on the immune response to infection, including microbial recognition, antimicrobial activities, and macrophage activation [29], it is likely that the early induction of ECMs plays a role in anti-*V. anguillarum* infection in flounder. Cytokines are essential regulators of inflammatory responses [30]. In our study, genes of cytokine–cytokine receptor interaction were found to be among the parental genes of *V. anguillarum*-induced DEcirs in flounder; however, this was not observed in *E. tarda*-induced DEcirs in flounder [16], suggesting that different pathogens induce different circRNA response in fish. During the process of infection, endocytosis acts to bring bacteria into the cell, thereby facilitating lysosome killing of the bacteria [31]. Consistently, in our study, endocytosis pathway was identified in the parental genes of DEcirs at 24 hpi. Cell adhesion molecules (CAMs) are known to exert crucial effects on immune cell function in inflammatory and infectious diseases by regulating cell to cell adhesion [32,33,34,35]. In our study, DEcirs-regulated CAMs were detected at all three time points of infection, suggesting an important role of these molecules in flounder immune defense against *V. anguillarum*.

Thirteen immune-related DEcirs were identified in this study, including circ_005398, circ_000857, circ_000859, and circ_004625. circ_005398 regulated the parental gene *SDC4*, which was involved in both CAMs and ECM receptor interaction pathways. In mammals, SDC4 is a cell surface receptor that functions in cell–ECM and cell–cell adhesion, antiviral signaling, and LPS-induced inflammation [36,37,38]. circ_000857 and circ_000859 were involved in cytokine–cytokine receptor interaction by targeting IL-4Rα, which was reported to control mice immune response to respiratory syncytial virus and *Leishmania* parasite infection [39,40]. circ_004625 was engaged in endocytosis through regulating SH3GLB2, an endophilin B family member that in mice is responsible for the endocytic response to influenza A virus infection by regulating the trafficking of endocytic vesicles and autophagosomes to late endosomes or lysosomes [41]. The detection of these immune-related DEcirs suggests a participation of these circRNAs in the immune response of flounder to *V. anguillarum* via the regulation of the parental genes.

Previous studies have shown that circRNAs can regulate host immunity by sponging miRNAs [42,43,44]. In our study, we found that one third of the DETmiRs in the immune-related DEcir-DETmiR networks were key immune miRNAs identified in a previous micro-transcriptome analysis *V. anguillarum*-infected flounder [23]. In the networks, circ_000857 and circ_000528 targeted miR-29-x, which was involved in the anti-pathogen response of half-smooth tongue sole, Nile tilapia, and zebrafish [45,46,47]. In addition, circ_000857 also targeted miR-146-x, which negatively modulated inflammatory response in humans and was active in patients with hepatitis B virus infection [48,49]. circ_000859 targeted nine DETmiRs, including miR-194-y, which was responsible for *E. tarda* escape from flounder immune defense [50], and miR-322-y, which was involved in *Spiroplasma eriocheiris*-induced apoptosis in mice [51]. Collectively, these results indicated that flounder circRNAs influenced pathogen-induced immune responses through interaction with miRNAs.

## 5. Conclusions

In this study, we systematically examined the circRNAs of flounder during *V. anguillarum* infection. A total of 148 DEcirs were identified, which were predicted to modulate parental genes involved in various pathways associated with immunity. Seven of the immune-related DEcirs also acted as miRNA sponges and targeted 18 DETmiRs, and the DEcirs and the DETmiRs formed regulatory networks. These results revealed an intimate implication of circRNAs in flounder immune response to *V. anguillarum* infection, and promoted our understanding of the roles of circRNAs in fish immunity against bacterial infection.

## Data Availability

The raw data of RNA sequencing and small RNA sequencing are available at the Sequence Read Archive (SRA) in NCBI with the accession numbers of PRJNA554220 and SRP241633, respectively. The datasets generated during this study are included in the article and its additional files.

## Supplementary Materials

The following are available online at https://www.mdpi.com/2073-4425/12/1/100/s1, Figure S1: Validation of the expression patterns of the parental genes of immune-related DEcirs by qRT-PCR, Figure S2: Validation of the expression patterns of the parental genes enriched in the ECM-receptor interaction pathway at 6 h post-infection (hpi) by qRT-PCR, Table S1: Summary of the primers used for qRT-PCR.
