# Supplementary material for: Systematic Identification and Analysis of Circular RNAs of Japanese Flounder (Paralichthys olivaceus) in Response to Vibrio anguillarum Infection
Source: Genes (Basel). 2021 Jan 15;12(1):100. doi: 10.3390/genes12010100 (PMC7830906; doi:10.3390/genes12010100)
Supplement: Supplementary file 1 [file genes-12-00100-s001.pdf]

Supplementary Materials:

Table S1. Summary of the primers used for qRT-PCR.

Name	Forward primer (5′ to 3′)	Reverse primer (5′ to 3′)
SDC4	GGACCGCTCTGTAGTTGGAC	CGTCTCCCTAACCTGCGACT
IL-4R	GAATTCACAACTCCTGCTGCC	GGAAAGTTGTCCCACGTCCT
CD276	CAAGCCTAGGAACCCAAGGA	GCTTTGGATTGTCTCCGAGC
H2-L	GATTCCGAATGGCAGCATCC	GCTTGGCACCGTGAGAATCA
TGFBR2	GAATGACAGTCTCTGGACGGG	GAAGGCGGCATAACAGCAGC
ITGA8	CTCTCTGCCAGCATAGACCG	CGCTCCACTCCGATGTAGTC
TUBA	TGACATCACAAACGCCTGCTTC	GCACCACATCTCCACGGTACAG

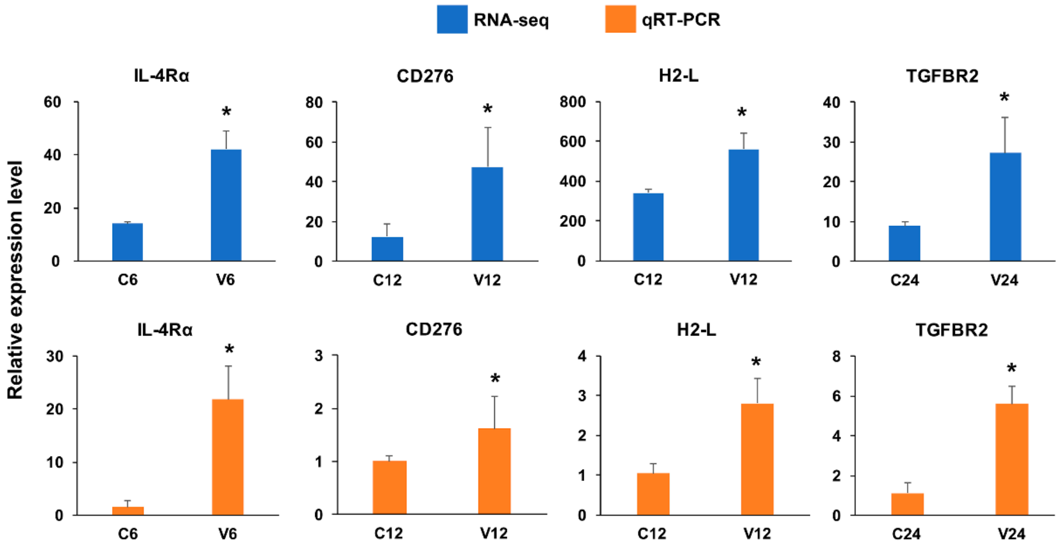

Figure S1. Validation of the expression patterns of the parental genes of immune-related DEcirs by qRT-PCR. Results are shown as means ± standard deviation (N = 3). *, *P* value < 0.05. For convenience, "C6", "C12", and "C24" indicate the control groups at 6, 12, and 24 h post-infection (hpi), respectively; "V6", "V12", and "V24" indicate the *V. anguillarum*-infected groups at 6, 12, and 24 hpi, respectively.

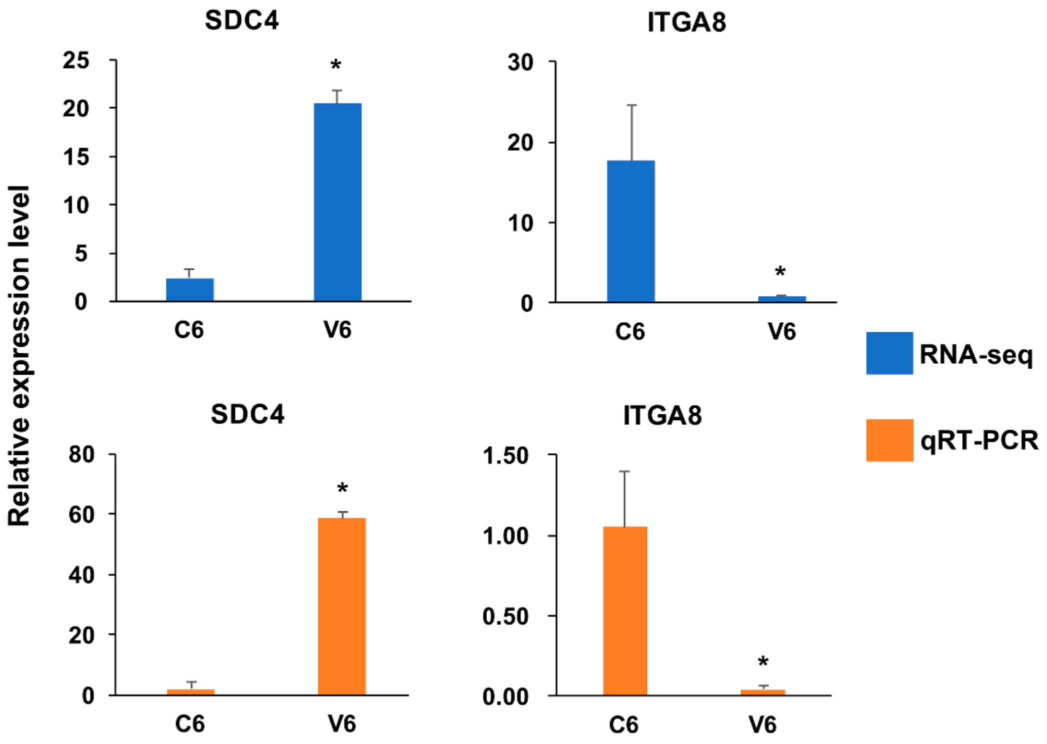

Figure S2. Validation of the expression patterns of the parental genes enriched in the ECM-receptor interaction pathway at 6 h post-infection (hpi) by qRT-PCR. Results are shown as means ± standard deviation (N = 3). *, *P* value < 0.05. For convenience, "C6" and "V6" indicate the control groups and the *Vibrio anguillarum*-infected groups, respectively.
